# Economic assessment of NGS testing workflow for NSCLC in a healthcare setting

**DOI:** 10.1016/j.heliyon.2024.e29272

**Published:** 2024-04-05

**Authors:** Davide Seminati, Vincenzo L'Imperio, Gabriele Casati, Joranda Ceku, Daniela Pilla, Carla Rossana Scalia, Gianluca Gragnano, Francesco Pepe, Pasquale Pisapia, Luca Sala, Diego Luigi Cortinovis, Francesca Bono, Umberto Malapelle, Giancarlo Troncone, Silvia Novello, Fabio Pagni

**Affiliations:** a Department of Medicine and Surgery, Pathology, Fondazione IRCCS San Gerardo dei Tintori, University of Milano-Bicocca, Monza, Italy; b Department of Public Health, Pathology, University of Naples Federico II, Naples, Italy; c Department of Medicine and Surgery, Oncology, Fondazione IRCCS San Gerardo dei Tintori, Monza, Italy; d Department of Oncology, University of Turin, Azienda Ospedaliero Universitaria San Luigi, Turin, Italy

## Abstract

**Background:**

The molecular diagnostic and therapeutic pathway of Non-Small Cell Lung Cancer (NSCLC) stands as a successful example of precision medicine. The scarcity of material and the increasing number of biomarkers to be tested have prompted the routine application of next-generation-sequencing (NGS) techniques. Despite its undeniable advantages, NGS involves high costs that may impede its broad adoption in laboratories. This study aims to assess the detailed costs linked to the integration of NGS diagnostics in NSCLC to comprehend their financial impact.

**Materials and methods:**

The retrospective analysis encompasses 210 cases of early and advanced stages NSCLC, analyzed with NGS and collected at the IRCCS San Gerardo dei Tintori Foundation (Monza, Italy). Molecular analyses were conducted on FFPE samples, with an hotspot panel capable of detecting DNA and RNA variants in 50 clinically relevant genes. The economic analysis employed a full-cost approach, encompassing direct and indirect costs, overheads, VAT (Value Added Tax).

**Results:**

We estimate a comprehensive cost for each sample of €1048.32. This cost represents a crucial investment in terms of NSCLC patients survival, despite constituting only around 1% of the expenses incurred in their molecular diagnostic and therapeutic pathway.

**Conclusions:**

The cost comparison between NGS test and the notably higher therapeutic costs highlights that the diagnostic phase is not the limiting economic factor. Developing NGS facilities structured in pathology networks may ensure appropriate technical expertise and efficient workflows.

## Highlights

• Cost Assessment: DNA/RNA NGS diagnostics in NSCLC - €1048.32 per sample.
• Financial Impact: Crucial investment, 1% of overall molecular expenses.
• Survival Investment: Enhances NSCLC patient survival rates.
• Economic Insight: Diagnostic phase is not the limiting factor.
• Optimization Recommendation: Develop NGS facilities in structured networks.

## 1 Introduction

Targetable genetic alterations are found in approximately 40–50% of Non Small Cell Lung Carcinomas (NSCLC), requiring specific predictive tests to appropriately identify patients for pharmacological therapies involving tyrosine kinase inhibitors (TKIs) [[1], [2], [3], [4]] [[1], [2], [3], [4]] [[1], [2], [3], [4]]. These treatments have resulted in unprecedented benefits in terms of Progression-Free Survival (PFS) and Overall Survival (OS) [5].

In this context, it is necessary to assess the molecular status of a minimal panel of so-called "must-test genes” [6]. Recently, lists of these mandatory genes have been compiled by various associations [[7], [8], [9], [10], [11]] [[7], [8], [9], [10], [11]] [[7], [8], [9], [10], [11]]. Additionally, in 2023, the European Society for Medical Oncology (ESMO) introduced un updated Scale for Clinical Actionability of Molecular Targets (ESCAT), providing a classification framework for molecular targets based on their clinical evidence of actionability (Supplementary table 1) [[12], [13], [14]] [[12], [13], [14]] [[12], [13], [14]]. Moreover, following the ADAURA and KEYNOTE-799 trials, the need for molecular analysis has expanded for EGFR and ALK genes from advanced stages to early stages too [15,16].

Next Generation Sequencing (NGS) technology offers a cost-effective solution with high sensitivity, adequate turn around time (TAT) and capable of analyzing multiple mutations [[17], [18], [19]] [[17], [18], [19]] [[17], [18], [19]]. NGS can be readily employed for whole-genome sequencing (WGS), whole-exome sequencing (WES), or hotspot regions in specific genes through the use of targeted sequencing panels (TS) [20]. The TS approach is usually the first choice in everyday practice, as it provides results more quickly, at lower costs, and with greater sensitivity [20]. For all these reasons, NGS platforms are increasingly used and recommended for conducting analysis of predictive biomarkers by guidelines [1,19,21]. In 2023, more than 16,000 NSCLC cases were molecularly analyzed in Italy, with more than 13,000 in the advanced stages [19,22]. Optimizing institutional workflows and implementing NGS facility networks could further increase the use of this method, reducing costs, TAT and improving patient care [23]. In this study, starting from a routine lab analytical economics, we aim to retrospectively perform a comprehensive microcost analysis for the execution of NGS diagnostics of NSCLC.

## 2 MATERIALS and methods

### 2.1 Population

**Fig. 1:**
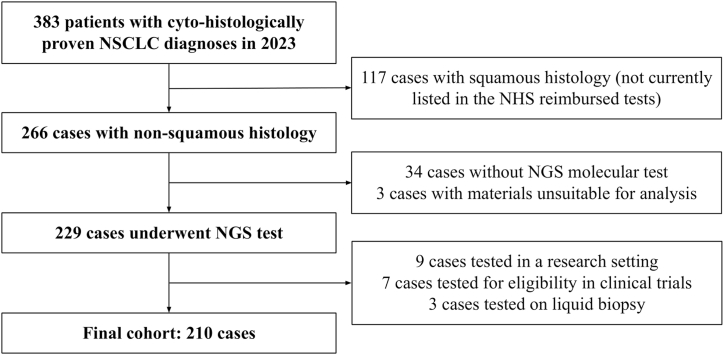
Flow diagram of the population enrollment process. NHS: National Health System.

**Fig. 2:**
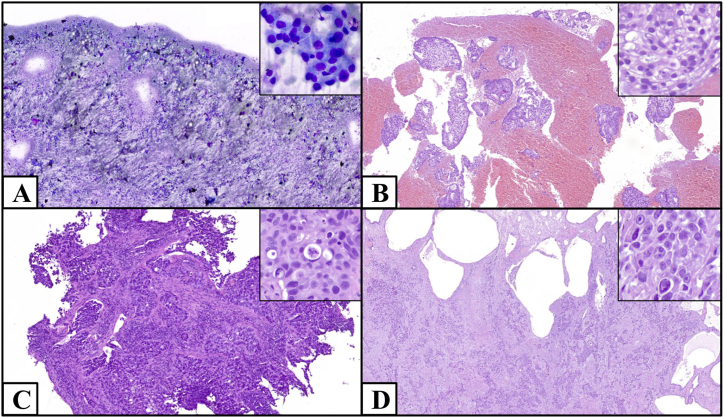
Examples of NSCLC samples on which pathological diagnoses and subsequent NGS analyses were performed. (A): air-dried cytological sample on a smear (MGG, x2, inset x40); (B): FFPE cytological sample, cell block (H&E, x4, inset x40); (C): biopsied histological sample (H&E, x5, inset x40); (D): histological sample from surgical resection (H&E, x2, inset x40).

We evaluated the molecular diagnostic workflow for the analysis of clinically relevant biomarkers in NSCLC samples, collecting data from January 1, 2023, to December 31, 2023 at the Oncological Molecular Pathology Unit of the IRCCS San Gerardo dei Tintori Foundation in Monza, Lombardy, Italy. Patients signed the institutional informed consent at the time of biopsy. Ethical Board approval was obtained (cod. 3585 RAS ATLAS, October 24, 2022). The study population encompasses all cyto-histological cases of non-squamous NSCLC that underwent NGS molecular testing within our Pathology Unit throughout the year 2023, regardless of tumoral stage and previous/concurrent known genetic variants (flow diagram reporting the inclusion/exclusion criteria for the cohort selection is summarized in Fig. 1). Cases with squamous histology or tested within a research/clinical trial setting were excluded due to the lack of specific tariff reimbursement for their characterization in NGS, according to the current regulations in the National Health Service (SSN, Servizio Sanitario Nazionale) of the Lombardy region [24]. Cases tested using liquid biopsy were excluded due to their notably higher NGS-related costs [25]. The decision to conduct the molecular test occurred only within a diagnostic and therapeutic framework and it was made by the clinicians managing the patients' care. Pathological diagnoses were made on routine cytological or histological samples (Fig. 2A–D).

### 2.2 NGS workflow

The automatic extraction and quantification of nucleic acids (DNA and RNA) was carried out using the Ion Torrent™ Genexus™ Purification System (GPI, Thermo Fisher Scientific, Waltham, MA, USA) with the Ion Torrent™ Genexus™ FFPE Combo Kit. Alternatively, extraction was performed using the Maxwell® CSC 16 Instrument (Promega, Madison, USA) with CSC DNA FFPE Kit or CSC RNA FFPE Kit, with nucleic acids quantification performed using the Qubit DNA dsDNA High Sensitivity Assay Kit and the Qubit RNA High Sensitivity Assay Kit on a Qubit 4.0 fluorimeter (Thermo Fisher Scientific). Templating and sequencing of all samples occurred on the Ion Torrent™ Genexus™ System platform (Thermo Fisher Scientific), which allows full automation of the workflow and enables simultaneous sequencing of DNA and RNA. The samples were sequenced using the GX5 chip. All samples were characterized using the Oncomine™ Precision Assay (OPA), capable of detecting 50 clinically relevant genes in NSCLC due to their presence in guidelines (21 genes) and/or clinical trials (48 genes) [26,27]. The computational analysis of sequencing data was performed using Ion Torrent Genexus Software version 6.6.2.1. Depending on clinical-interpretative needs, some NGS results were confirmed using orthogonal methods, such as immunohistochemistry (IHC, ALK clone D5F3 and BRAF clone VE1 on the Dako Omnis platform, Glostrup, Denmark) or real-time PCR (EasyPGX qPCR instrument 96, EasyPGX ready ALK/ROS1/RET/MET kit, Diatech Pharmacogenetics, Jesi, Italy).

### 2.3 Costs Estimation

To determine the unit cost per sample for each test, we applied a full cost approach, which is determined by the sum of direct costs, indirect costs, overheads, and VAT (Value Added Tax). The direct production costs include staff labor and healthcare consumption (consumable materials), while indirect production costs consist of healthcare equipment and common costs of the facility (overheads). The overheads encompass maintenance, utilities, cleaning, non-healthcare materials, management, accounting, etc., and are fixed at 25% of the sum of the aforementioned costs, according to standard institutional policies [6,28] [[6], [28], [29], [30]] [[6], [28], [29], [30]].

**Fig. 3:**
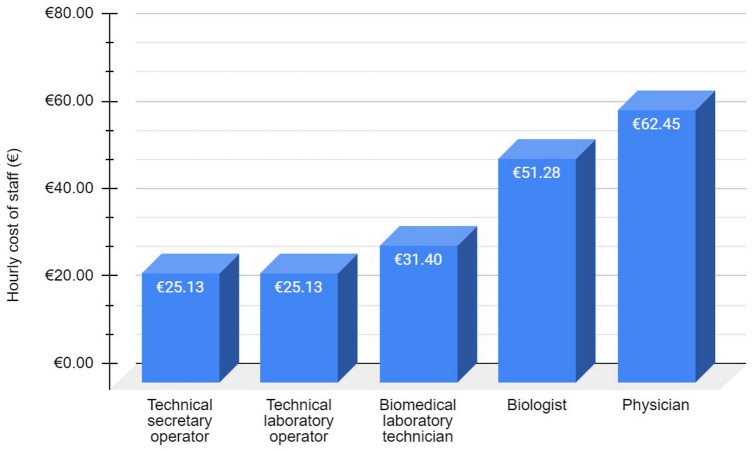
Gross hourly wage of the staff.

To determine the labor cost, the gross hourly wage of the staff was resumed by the hospital's supply department (Fig. 3). The cost of consumables per single sample was calculated assessing the number of samples that can actually be processed for each component (reagent saturation). The number of samples per run (machine saturation) for each method was calculated, taking into account possible positive and/or negative controls. The annual cost of equipment, including purchase price, maintenance, and technical assistance, was evaluated over 12 months of activity, regardless of the tumor sample, considering - as per the literature - a depreciation period of 5 years [28]. The costs of microscopes, microtomes, water baths, cooling plates, IHC platform, and nucleic acid quantification were not included as they are negligible due to the high number of annual samples processed and their different depreciation period, usually set at 10 years [30,31].

The cost analysis of TKIs therapy was based on the prices currently in effect at the hospital pharmacy (Table 1). This analysis was performed for all advanced-stage NSCLC harboring ESCAT I-II variants with drugs approved by EMA (European Medicines Agency) and reimbursed by AIFA (Agenzia Italiana del Farmaco), in the first and/or second-line treatment, at standard dosage and without treatment interruptions, in relation to median data for PFS [12,[32], [33], [34]]. For the second-line therapy (Lorlatinib, Tepotinib), the percentage of patients actually treated - according to the literature data - was taken into account [35,36].

**Table 1:** Monthly cost of each targeted therapy (VAT included).

Gene	Therapy	Dosage	Monthly cost (€)
ALK	Alectinib	1200 mg/day	4027.2
	Lorlatinib	100 mg/day	2970
BRAF	Dabrafenib + Trametinib	300 mg/day +2 mg/day	5850
EGFR	Osimertinib	80 mg/day	3704.1
MET	Tepotinib	450 mg/day	4581.6
ROS1	Entrectinib	600 mg/day	5236.2

### 2.4 Statistical analysis

All costs are presented in euros (€) of 2023 and include VAT, which in Italy is currently set at 10% for drugs and 22% for reagents and platforms [28]. Costs related to participation in external quality assessment programs (VEQ) were excluded from the analysis. Data analysis was performed using Excel software (Microsoft, Redmond, USA). Where appropriate, data were rounded to the nearest decimal place.

## 3 RESULTS

### 3.1 Population

The case series comprises 210 cases of non-squamous NSCLC, with 78.1% of them in advanced stages (stages IIIB-IIIC-IVA-IVB according to AJCC eighth edition) (Supplementary table 2) [37].

### 3.2 Extraction and molecular results

**Fig. 4:**
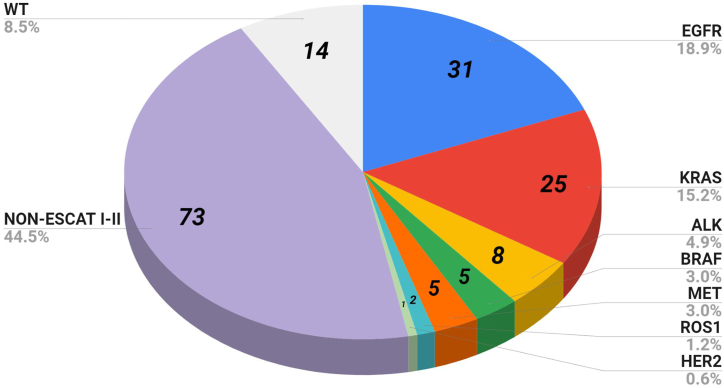
Pie chart of ESCAT I-II variants detected in advanced stage. WT: wild-type. ESCAT: ESMO Scale for Clinical Actionability of Molecular Targets.

The results of nucleic acid extraction and NGS tests are summarized in Fig. 4 and Supplementary table 3. A subset of cases (10%) was confirmed using orthogonal methods, including IHC (66%, 86% for ALK evaluation) and/or real-time PCR (38%, 75% for MET exon 14 skipping assessment). A small proportion of cases (3.8%) underwent NGS reanalysis due to pre-analytical, analytical, or post-analytical issues. The average TAT was 9.89 working days. The analysis of the actual hands-on time and the total staff cost per individual sample for each method used is summarized in Table 2.

**Table 2:** Actual hands-on time and total personnel cost per individual sample in each method used. IHC: immunohistochemistry; GPI: Ion Torrent Genexus Purification System; PCR: polymerase chain reaction; NGS: Next Generation Sequencing.

	Staff time (minutes)					Staff cost per sample (€)
Service	Technical secretary operator	Technical laboratory operator	Biomedical laboratory technician	Biologist	Physician	
**IHC**	–	10	10	–	5	**14.61**
**Extraction Maxwell**	5	10	5	–	–	**8.88**
**Extraction GPI**	5	10	5	–	–	**9.40**
**real-time PCR**	–	–	30	10	10	**34.64**
**NGS**	–	–	5	20	20	**35.58**

### 3.3 Cost analysis

Annualized cost of the platforms is reported in Supplementary table 4, including the purchase price, maintenance and technical support, weighted against the total number of samples processed by the platform in 1 year. At the conclusion of the analyses, the costs for each test are as follows: €1048.32 for NGS, €198.06 for real-time PCR, and €68.26 for IHC (Table 3). The pharmacological cost of targeted therapy in patients with advanced-stage NSCLC with ESCAT I-II variants, using drugs approved by EMA and reimbursed by AIFA, in the first and/or second-line treatment (52 cases), at standard dosage and without treatment interruptions, is shown in Supplementary table 5.

**Table 3:** Cost in euros (€) for predictive tests per single sample. IHC: immunohistochemistry. PCR: polymerase chain reaction. NGS: Next Generation Sequencing. FFPE: formalin fixed, paraffin embedded. VAT: Value Added Tax.

Cost categories	IHC	real-time PCR	NGS FFPE	% ratio
Staff labor	14.61	25.17	35.58	5.7
Reagents	40	73.2	563.38	51.4
Platform	marginal	34.09	209.52	18.5
Extraction	/	25.99	30.18	4.3
Overheads	25%			
VAT	22%			
**Total**	**68.26**	**198.06**	**1048.32**	

## 4 Discussion

The recent improvement in NSCLC patients survival is a direct result of the increase in the number of targetable biomarker genes and available targeted drugs [5,38]. The ESMO ESCAT scale has recently provided an updated classification of molecular targets for NSCLC based on clinical evidence of gene variants actionability [12]. The implementation of personalized therapeutic treatments, according to the precision medicine model, is primarily possible through the use of NGS tests, characterized by automation, high sensitivity, and the ability to provide the genetic characterization of numerous biomarkers in a short time frame, across multiple tumor types, and simultaneously for multiple patients [39]. NGS use has also demonstrated a survival advantage, as it allows for extensive analysis in relatively short times, making targeted therapy available to a broader range of patients [32,40,41]. However, NGS platforms have not been universally adopted by all diagnostic laboratories, primarily due to the high level of expertise required for staff, spanning knowledge in medicine, biology, genetics, and bioinformatics, as well as the significant costs associated with setting up the laboratory and conducting tests [1].

To understand the actual financial needs associated with molecular diagnostic activities, we analyzed the costs related to our case study of 210 NSCLC samples collected from January 01, 2023 to December 31, 2023. The total NGS testing cost for the simultaneous DNA and RNA analysis of 50 genes, including VAT (22%), direct and indirect expenses, and overheads necessary for the test execution (nucleic acid extraction, consumables, sequencing platform, maintenance, utilities, non-medical materials, labor, etc.), amounted to €1048.32 for solid samples (FFPE cytological/histological specimens). The cost of orthogonal additional analyses and NGS reanalyses, distributed across the sample size, led to a 4.9% increase in the cost per sample, bringing the final cost of NGS testing to €1100.35 per patient.

It is often argued in the literature that using NGS for NSCLC biomarker analysis provides an economic advantage - in addition to the diagnostic one - compared to the "single-gene testing" (SGT), relying on IHC, real-time PCR, FISH, and Sanger sequencing [6,28,42,43]. Traditionally, the SGT is considered labor-intensive, with a narrower reference range and variable TAT due to the sequential analysis steps [[17], [18], [19], [28], [32], [34], [44]] [[17], [18], [19], [28], [32], [34], [44]] [19,28,32,34,44]. However, the NGS economic advantage would occur despite its elevated unit cost if compared to the SGT methods, having in our experience +1435% and +429% higher costs compared to IHC and real-time PCR, respectively. This is comparable to what is reported in the literature, owing to the possibility in NGS to simultaneously test all biomarkers of interest [6,44]. In particular, we found that the most significant cost increase between real-time PCR and NGS is observed in the categories of reagents (+670%) and platform (+461%). Recently, an italian study compared the economic burden of testing the main biomarkers in advanced NSCLC using both diagnostic approaches (SGT and NGS), estimating an average cost per patient of €1203 with SGT and €643 with NGS [28]. In conclusion, it was hypothesized that NGS could lead to savings of up to €522 per patient, depending on the number of biomarkers studied and the platform used [28]. The economically advantages for NGS, albeit with relatively negligible savings (€25), would start with the analysis of 3 biomarkers, an approach that is currently limiting in advanced stages and more suitable for postoperative evaluation of early stages [28]. Similarly, another Italian study estimated that the average cost per SGT and NGS would be €3055 and €1,402, respectively, highlighting that NGS economic advantage would still start from the analysis of the third biomarker [6]. Other studies considering more genes determined NGS overall savings ranging from €25 to €1041 per patient, in addition to recognizing an advantage in terms of reduced depletion of tumor sample, TAT, and overall hands-on time [28,45] [[28], [45], [46], [47]] [[28], [45], [46], [47]]. NGS appears to be particularly advantageous as the number of biomarkers to be tested increases, suggesting that the progressive growing number of targetable molecular alterations will probably increase the potential savings achieved with NGS [6,28]. In 2020, a Brazilian study estimated that an investment of approximately €3250 in NGS testing is required to correctly identify a case of NSCLC with alterations in EGFR, ALK, or ROS1 (3 biomarkers) [48].

In our experience, it was necessary to invest €2232 to identify an ESCAT I-II variant (currently 9 biomarker genes in advanced NSCLC), while - considering only the same 3 biomarkers - the cost would have been €4,193, confirming the hypothesis that as the number of genes to be tested increases, the cost per identified alteration decreases [44]. Evaluating only cases with targeted therapy approved by EMA and reimbursed by AIFA (5 biomarker genes), the cost for each targetable alteration identified was €3371. Applying the sequential SGT approach would have resulted in a cost per patient ranging from €198 to €1058 (average €511), with NGS providing a small economic saving (€10) starting from the analysis of the sixth biomarker (23.7% of cases). The total cost incurred with SGT would have been €83,930 compared to the higher €171,924 with NGS (+104%). The cost per ESCAT I-II variant identified in SGT scenario would have decreased from €2232 to €1090 (−51%), and that for variants with reimbursed therapy from €3371 to €1645 (−51%). On the other hand, SGT would lead to a significant increase in hands-on time, leading the total working hours for a healthcare technician from 17.5 to 231.5 (+1223%) and for both biologist and physician from 70 to 77 (+10%). However, in the precision medicine era, facing an increasingly complex and personalized diagnostic-therapeutic scenario, the approximate data provided by SGT methods, which are often incapable to indicate the precise genetic variant of the patient, with lower sensitivity and reference ranges and also requiring more genomic material input, making patients prone to a higher risk of rebiopsy exacerbating TAT, should prompt the use of more advanced and precise analyses [6,32,43,[49], [50], [51], [52], [53]]. Furthermore, NGS provides an extensive characterization of the tumor biology, leading to the identification of variants that could benefit the patient from dedicated clinical trials, detecting co-mutations, or precisely recognizing fusion partners, thus better stratifying the response to therapy [19,43].

The strong difference in the cost of NGS testing in different hospital centers is well highlighted by Pisapia et al. study, in which is illustrated how NGS test cost with similar gene panels varied from a minimum of €394 to a maximum of €2,676, according to the center where it was performed and the retest rate, with a maximum delta of €2,282, which remains essentially constant even with a broader gene panel [6]. However, it should be noted that methodological heterogeneity, differences in costs between different countries, and the frequent lack of sufficient data transparency make direct comparisons complex, issues already raised by some authors on the subject, who advocate for the development of dedicated guidelines for this type of analysis [28,[54], [55], [56]].

Evaluating the therapeutic cost in light of the expected median PFS for each targeted treatment, we estimate an average cost of €74,363 per patient, in contrast to a much lower cost for molecular testing of €1,048, with a diagnosis/treatment ratio of 1.4%–98.6%, without considering additional cost items such as hospitalization costs, staging and follow-up tests, management of adverse events (AE), clinical staff, etc. Other studies also described that molecular/predictive diagnostic costs represent approximately 1% of the overall therapeutic costs, regardless of the method used and the type of cancer under examination [34,57].

Our analyses were performed with an average TAT of 9.89 days and a median of 10 days, consistent with the literature data [18,34]. The execution of orthogonal confirmation methods or the repetition of NGS analysis did not have a particularly significant impact on TAT, as their exclusion from the calculation resulted in a decrease in the average TAT from 9.89 to 9.44 days, confirming the hypothesis that the cases collection time is the true time-limiting step in the molecular diagnostic workflow.

This work has some limitations. First, the analysis is retrospective and represents what happens in a single medium-sized Italian center in terms of number of molecular tests performed. Second, the targeted therapy cost analysis is limited to ESCAT I-II biomarkers with currently EMA-approved drugs reimbursed in Italy and it has been calculated on the assumption that all patients would take the therapy at standard dosages without interruptions. Lastly, being unicentric it does only partly take into account the possible variability of equipment unit costs, which can be influenced by the specific setting and over time, being often strictly dependent on tender procedures and procurement processes. This study does not aim to conduct a cost-effectiveness analysis.

## 5 Conclusions

The total NGS testing cost for the simultaneous DNA and RNA analysis of 50 genes, including VAT, direct and indirect expenses, and overheads necessary for the test execution, amounted to €1048 for solid samples. The significantly higher therapeutic costs highlight that the diagnostic phase, a critical point in the patient's care pathway for NSCLC, is not the economically limiting factor in precision medicine. Building a well-structured molecular network tailored to regional needs and supported by adequate technical expertise could be the key to an effective and sustainable NGS testing.

## Authors’ contributions

DS defined the study design, performed the retrospective research and data extraction, as well as the elaboration of the computational pipeline for the study. DS, GCA, JC, DP and CS performed molecular analyses. DS and GCA conducted interpretation of NGS data. GGR, FPE, PP, GT and UM provided counseling as experts in molecular pathology. LS, DLC, FB and SV provided counseling as experts in lung pathology. FPA and VL performed the supervision of the work, revising critically the manuscript before the approval by all the authors. FPA provided the funding acquisition and administrative support. All authors were involved in writing the paper and had final approval of the submitted and published versions.

## Ethical consideration

The research was performed in accordance with the requirements of the World Medical Association's Declaration of Helsinki. Ethical Board approval was obtained (cod. 3585 RAS ATLAS, October 24, 2022).

## Data Availability

Data are available upon reasonable request to the corresponding author.

## Funding

The present work has been funded by the Italian Ministry of the University MUR Dipartimenti di Eccellenza 2023–2027 (l. 232/2016, art. 1, commi 314–337).

## CRediT authorship contribution statement

**Davide Seminati:** Writing – original draft, Visualization, Validation, Supervision, Software, Resources, Project administration, Methodology, Investigation, Formal analysis, Data curation, Conceptualization. **Vincenzo L'Imperio:** Writing – original draft, Visualization, Validation, Supervision, Resources, Methodology, Investigation, Formal analysis, Data curation, Conceptualization. **Gabriele Casati:** Methodology, Formal analysis, Data curation, Conceptualization. **Joranda Ceku:** Formal analysis, Data curation. **Daniela Pilla:** Formal analysis, Data curation. **Carla Rossana Scalia:** Formal analysis, Data curation. **Gianluca Gragnano:** Conceptualization. **Francesco Pepe:** Conceptualization. **Pasquale Pisapia:** Conceptualization. **Luca Sala:** Conceptualization. **Diego Luigi Cortinovis:** Conceptualization. **Francesca Bono:** Conceptualization. **Umberto Malapelle:** Conceptualization. **Giancarlo Troncone:** Conceptualization. **Silvia Novello:** Conceptualization. **Fabio Pagni:** Writing – original draft, Visualization, Validation, Supervision, Software, Resources, Project administration, Methodology, Investigation, Funding acquisition, Formal analysis, Data curation, Conceptualization.

## Declaration of competing interest

VL received personal fees (as consultant and/or speaker bureau) from Eli Lilly, Roche, Novartis. PP has received personal fees as speaker bureau from Novartis. UM has received personal fees (as consultant and/or speaker bureau) from Boehringer Ingelheim, Roche, MSD, Amgen, Thermo Fisher Scientific, Eli Lilly, Diaceutics, GSK, Merck, AstraZeneca, Janssen, Diatech, Novartis, and Hedera. GT reports personal fees (as speaker bureau or advisor) from Roche, MSD, Pfizer, Boehringer Ingelheim, Eli Lilly, BMS, GSK, Menarini, AstraZeneca, Amgen, and Bayer. DLC has received personal fees (as consultant and/or speaker bureau) from Boehringer Ingelheim, Roche, MSD, Amgen, Eli Lilly, GSK, Merck, AstraZeneca, Janssen, Novartis. Silvia Novello received personal fees (as consultant and/or speaker bureau) from Sanofi, AstraZeneca, MSD, Bristol-Myers Squibb, Roche, Pfizer, Lilly, Takeda, AbbVie, Boehringer Ingelheim, Bayer, Amgen, BeiGene, Novartis, Janssen. FP has received personal fees (as consultant and/or speaker bureau) from Novartis, Roche, MSD, Amgen, GSK, AstraZeneca, Lilly, LogiBiotech, Diapath. All fees received by the author are unrelated to the current work.
