# Supplementary material for: Economic assessment of NGS testing workflow for NSCLC in a healthcare setting
Source: Heliyon. 2024 Apr 5;10(7):e29272. doi: 10.1016/j.heliyon.2024.e29272 (PMC11015456; doi:10.1016/j.heliyon.2024.e29272)
Supplement: Multimedia component 1 [file mmc1.docx]

**SUPPLEMENTARY TABLES**

| **Gene** | **Therapy** | **ESCAT tier** |
| --- | --- | --- |
| ALK | Alectinib, Brigatinib, Lorlatinib | I-A |
|  | Ceritinib, Crizotinib | I-B |
| BRAF | Dabrafenib + Trametinib | I-B |
| EGFR | Afatinib, Dacomitinib, Erlotinib, Erlotinib-Bevacizumab, Gefitinib, Osimertinib | I-A |
|  | Gefitinib-Carboplatino-Pemetrexed | I-B |
| EGFR ex20ins | Amivantamab, Mobocertinib | I-B |
| HER2 | Trastuzumab deruxtecan | II-B |
| KRAS | Adagrasib, Sotorasib | I-B |
| MET | Capmatinib, Tepotinib | I-B |
| NTRK | Entrectinib, Larotrectinib | I-C |
| RET | Pralsetinib, Selpercatinib | I-C |
| ROS1 | Crizotinib, Entrectinib, Repotrectinib | I-B |

# **Supplementary Table 1**: Genes with ESCAT I-II variants in NSCLC and their EMA-approved drugs (European Medicines Agency, 2023). ESCAT: ESMO Scale for Clinical Actionability of Molecular Targets[^12^](https://paperpile.com/c/bSb7QQ/kelt4).

| **Cohort data** | | |
| --- | --- | --- |
| **N°** | 210 | |
| **Age (years)** | Min | 38 |
|  | Max | 88 |
|  | Average | 68,9 |
| **Sex** | M | 112 (53.3%) |
|  | F | 98 (46.7%) |
| **Stage (1)** | Early | 46 (21.9%) |
|  | Advanced | 164 (78.1%) |
| **Histotype** | Adenocarcinoma | 183 (87.1%) |
|  | Non-squamous carcinoma | 21 (10%) |
|  | Adenosquamous carcinoma | 3 (1.4%) |
|  | Pleomorphic carcinoma | 3 (1.4%) |
| **Specimen type** | Biopsy | 80 (38.1%) |
|  | Cytoinclusion | 70 (33.3%) |
|  | Resection | 58 (27.6%) |
|  | Smear | 2 (1%) |
| **Site of collection** | Primary tumor | 111 (52.9%) |
|  | Metastasis | 99 (47.1%) |

# **Supplementary Table 2**: Patient cohort data. (1) According to the AJCC eighth edition guidelines[^37^](https://paperpile.com/c/bSb7QQ/gXfLM).

| **Molecular results** | | | |
| --- | --- | --- | --- |
| **Neoplastic cellularity (%)** | Min | 10 | |
|  | Max | 90 | |
|  | Median | 50 | |
|  | Median resection | 50 | |
|  | Median other sample types | 55 | |
| **Extraction (ng/mL)** | Nucleic acids | DNA | RNA |
|  | Min | 0.14 | 0.53 |
|  | Max | 341.8 | 620.67 |
|  | Median | 8.03 | 23.58 |
| **Extraction TNA (average, ng/mL)** | Biopsy | 24.93 | |
|  | Cytoinclusion | 12.89 | |
|  | Resection | 150.45 | |
| **ESCAT I-II variants** | N° | 86 (40.9%) | |
|  | Early | 9 (19.6%) | |
|  | Advanced | 77 (46.9%) | |
| **Clinically relevant**  **variants** | N° | 193 (91.9%) | |
|  | Early | 43 (93.5%) | |
|  | Advanced | 150 (91.5%) | |
| **Wild-type** | N° | 17 (8.1%) | |
|  | Early | 3 (6.5%) | |
|  | Advanced | 14 (8.5%) | |

# **Supplementary Table 3**: Nucleic acid extraction and NGS test results. TNA: total nucleic acids. ESCAT: ESMO Scale for Clinical Actionability of Molecular Targets[^12^](https://paperpile.com/c/bSb7QQ/kelt4).

| **Platform** | **Annual cost (5-year amortization)** | **Number of samples in 2023** | **Platform cost per sample** |
| --- | --- | --- | --- |
| **Maxwell CSC 16 Instrument** | 5,340 | 884 | **6.04** |
| **EasyPGX qPCR Instrument 96** | 6,000 | 176 | **34.09** |
| **Ion Torrent Genexus System** | 102,245.96 | 488 | **209.52** |

# **Supplementary Table 4**: Annual platform total cost and cost per sample.

#

| **Gene (1)** | | **1st line therapy** | **Monthly cost of 1st line therapy (€)** | **1st line PFS** | **Estimated total cost of 1st line therapy (€)** | **2nd line therapy** | **Monthly cost of 2nd line therapy (€)** | **2nd line PFS** | **Estimated total cost of 2nd line therapy (€)** | **% of patients treated in 2nd line** | **Total therapy cost per patient (€)** | **Number of patients in advanced stage** | **Total targeted therapy cost (€)** |
| --- | --- | --- | --- | --- | --- | --- | --- | --- | --- | --- | --- | --- | --- |
| ALK | | Alectinib | 4,027.2 | 34.8 | 140,146.56 | Lorlatinib | 2,970 | 6.2 | 18,414 | 40% | 147,512.16 | 8 | 1,180,097.28 |
| BRAF p.V600 | | Dabrafenib + Trametinib | 5,850 | 10.8 | 63,765 | - | - | - | - | - | - | 5 | 318,825 |
| EGFR | common | Osimertinib | 3,704.1 | 18.9 | 70,007.49 | - | - | - | - | - | - | 28 | 1,960,209.72 |
|  | uncommon |  |  | 8.2 | 30,373.62 | - | - | - | - | - | - | 3 | 91,120.86 |
| MET ex14 skipping | | - | - | - | - | Tepotinib | 4,581.6 | 8.5 | 38,943.6 | 40% | 15,577.44 | 5 | 77,887.2 |
| ROS1 | | Entrectinib | 5,236.2 | 15.7 | 82,208.34 | - | - | - | - | - | - | 2 | 164,416.68 |
|  |  |  |  |  |  |  |  |  |  |  |  | 51 | 3,792,556.74 |

# **Supplementary Table 5**: Pharmacological cost of targeted therapy. (1): Genes harboring ESCAT I-II variants with drugs approved by EMA and reimbursed by AIFA. PFS: Progression Free Survival, median, in months.
